# Micro-Vibration-Based Slip Detection in Tactile Force Sensors

**DOI:** 10.3390/s140100709

**Published:** 2014-01-03

**Authors:** Raul Fernandez, Ismael Payo, Andres S. Vazquez, Jonathan Becedas

**Affiliations:** 1 Department of Electrical, Electronics and Control Engineering, University of Castilla-La Mancha, Ciudad Real 13071, Spain; E-Mails: andress.vazquez@uclm.es (A.S.V.); jonathanbecedas@hotmail.com (J.B.); 2 Department of Electrical, Electronics and Control Engineering, University of Castilla-La Mancha, Toledo 45071, Spain; E-Mail: ismael.payo@uclm.es

**Keywords:** tactile sensors, slipping, grasping, manipulation

## Abstract

Tactile sensing provides critical information, such as force, texture, shape or temperature, in manipulation tasks. In particular, tactile sensors traditionally used in robotics are emphasized in contact force determination for grasping control and object recognition. Nevertheless, slip detection is also crucial to successfully manipulate an object. Several approaches have appeared to detect slipping, the majority being a combination of complex sensors with complex algorithms. In this paper, we deal with simplicity, analyzing how a novel, but simple, algorithm, based on micro-vibration detection, can be used in a simple, but low-cost and durable, force sensor. We also analyze the results of using the same principle to detect slipping in other force sensors based on flexible parts. In particular, we show and compare the slip detection with: (i) a flexible finger, designed by the authors, acting as a force sensor; (ii) the finger torque sensor of a commercial robotic hand; (iii) a commercial six-axis force sensor mounted on the wrist of a robot; and (iv) a fingertip piezoresistive matrix sensor.

## 1. Introduction

During the last 30 years, robots have evolved to interact with the environment. One major issue that was studied for that purpose was the artificial sense of touch, widely known as tactile sensing [1].

Tactile sensing allows the robots to physically interact with the environment by improving basic capabilities, such as touching [2], detecting surfaces and collisions [3,4] and advanced capabilities, such as grasping [5–7] and the manipulation of objects [8–10].

To carry out the previously mentioned basic and advanced skills with robots, scientists have designed specific tools. The most popular are grippers and anthropomorphic hands [11]. However, they require one to have accurate and sophisticated force and torque sensors installed to develop the previously mentioned skills. Those sensors replicate human finger tactile perception to obtain information about the applied forces exerted over an object and its shape. In addition, solving complex kinematics and dynamics systems is required to obtain information about the interaction with the environment [8,10,12–15]. However, the usefulness of tactile sensing has provided good results in other scientific areas and applications: medicine, rehabilitation, prostheses, virtual reality, the food industry and industrial automation [9,16–19].

In addition to the complexity of the tactile interaction previously explained, the sensors commonly used and developed in the previous citations have limitations when the environment presents uncertainty. In this case, sensing is combined with complex and non-deterministic control strategies to obtain more relevant information about the contact [20], mainly in grasping, manipulation and examination of objects, in which high accuracy in positioning and applied forces is required.

Some approaches have been proposed to create more sophisticated tactile sensors that can deal with unstructured situations and tactility. They are based on piezoresistive, capacity, optical and piezoelectrical transductive technologies [16,21]. However, they have limited working range, limited spatial resolution and a high cost.

Other approaches did not develop new sensing technologies, but combined different types of tactile sensors [12,14]. However, the complexity of the mechanical system was also high, as well as the cost.

Nevertheless, knowledge of the environment can be increased so as to improve the performance of the sensors by properly designing sensors with existing and cost-effective technology. At the end of the 1980s, it was demonstrated that slip detection highly contributes to increasing knowledge over uncertainty, and it improves manipulation without increasing the complexity of the sensors and control systems [22].

### 1.1. Slip Detection

In addition, from human physical experience, we know that avoiding slipping is crucial to successfully manipulate an object. However, specific tactics to detect and avoid slipping have not been extensively used; most of the studies focus on applying a sufficiently high and previously known force to avoid it [7]. However, the early detection of slipping can contribute to controlling the contact forces to prevent slipping and to successfully manipulate an uncertain object.

Different sensors to detect slipping can be found in the scientific literature:

(i) The use of specifically designed skin sensors: In [22], the fingers of a robotic system were covered with foam rubber, and this was covered with rubber skin. Between the foam and the rubber, accelerometers were strategically disposed to measure the accelerations that the skin suffered when it was in contact with a slipping surface. They observed that low amplitude, high frequency vibrations were produced in the finger skin when the contact surface was slipping. In [23], a grasping force control to avoid slipping based on the estimation of the surface's friction coefficients was implemented in a similar system. However, the accelerometers were really noisy and affected the measurements of small amplitude signals, so there was a systematic threshold under which the slipping could not be detected. Furthermore, those sensors could not measure the location of the contact point, which is useful for grasping unknown objects. In [24,25], silicon rubber was used to create a flexible skin sensor, in which numerous strain gauges were linearly distributed. The slipping between surfaces was detected by analyzing the deformation pattern of the strain gauges. In that work, both slipping and the location of the slipping point in the sensor were estimated, but not directly measured. Although promising results with strain gauges were obtained, they have not been widely studied for slip detection. Furthermore, skin sensors made of flexible materials, such as foam or rubber, are expensive, because they are of a dedicated designed; they are not durable; they are not resistant to exposure to high temperature surfaces, rough surfaces or surfaces with cutting or pointing edges.
(ii) Use of array/matricial sensors: In [26], a designed 16 × 16 matrix of conductive rubber was used. Slipping was detected by interpreting the deformation of the contact surface based on the change of the center of mass of the contact surface and by measuring the vibration produced in the skin when slipping occurred. The resolution for localizing the object was poor. In [27], a tactile array sensor was combined with a dynamic sensor to accurately detect the location of the object and the slipping effect by measuring the micro-vibrations caused by slipping. Analogous to previous work, Zhang and Liu propose in [28] a method based on the vibration analysis of the pressure center of array-type sensors.
(iii) Combination of sensors: Other approaches used a set of sensors to detect slipping, such as vision combined with tactile sensors [29] or a silicon rubber-based touch sensor combined with force sensors [30]. In [31], a conductive film sensor was used to detect slipping by studying the pressure load of the contact. In [32], a combination of tactile array sensors and force/torque sensors based on strain gauges was used for slip detection.

As a consequence, slipping can be detected by using three techniques:

(i) Estimating the friction coefficient between the grasping surfaces, which is not an accurate indirect measurement.
(ii) Analyzing the changes in the contact footprint over a tactile sensor: changes in the shape of the contact with the object and changes in the pressure-force distribution. This strategy is not useful if the object is not rigid.
(iii) Detecting micro-vibrations. This is a direct and reliable measure of slipping, but it is of a dynamic nature. Therefore, the dynamic sensors traditionally used, such as accelerometers, are noisy and usually detect slipping too late to respond to it and to prevent it with a closed loop control law.

### 1.2. Our Approach

In this paper, we design and test a simple and low-cost sensor that can be used in different applications and incorporated in robotic hands for grasping and manipulation tasks when the environment presents uncertainty.

The sensor was geometrically designed to detect the contact location with high accuracy and to be sensitive to the micro-vibrations produced in the contact surfaces when slipping occurs. It is based on the strategic location of strain gauges in an elastic concentrator covered by a semi-cylindrical metallic capsule. Thus, the sensor plate is separated from the contact surface. This contributes to increasing the life of the sensor and protecting it from accidental and dangerous contacts (high temperature surfaces, accidental hits and cutting surfaces, among others, in contrast to rubber or foam surfaces).

Furthermore, strain gauges present the following advantages: high load range, high precision, high resolution, low hysteresis, high sensitivity, linear response and low cost [16].

Our method's concept is similar to other methods that analyze microvibration at a high frequency, which excitates the tactile sensors when a slipping contact occurs [13,26,30]. The novelty of our method lies in our combination of well-known signal processing principles, discrete Fourier transform (DFT), fast Fourier transform (FFT) and power spectrum (PS), in order to create a real-time slip detection algorithm.

The sensor performance in slip detection is analyzed and compared with a flexible finger acting as a force sensor (designed by the authors), the finger torque sensor of a commercial robotic hand (BarrettHand), a commercial six-axis force sensor mounted in the wrist of a robot and a fingertip piezoresistive commercial matrix sensor. The slip detection algorithm is implemented in all the sensors to test its performance and feasibility.

The paper is structured with six sections. Section 2 deals with the description of the tactile sensor. In Section 3, we present our slip detection algorithm. In Section 4, some experimental tests are shown. In Section 5, we present slip detection with other sensors, and finally, in Section 6, the main conclusions of this work are summarized.

## 2. Description of the Tactile Sensor

**Figure 1.**
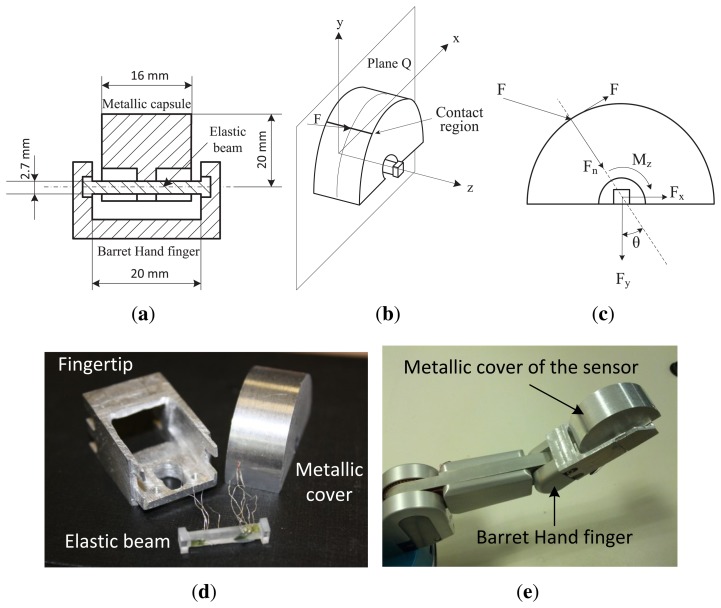
(**a**) Cross-section of the sensor; (**b**) applied force; (**c**) decomposition of force *F;* (**d**) sensor parts; (**e**) sensor mounted on a BarrettHand finger.

The sensor is constituted by two structural parts, as shown in Figure 1d: (1) the passive part (a semi-cylindrical metallic cover of aluminum), which comes into contact with the object; and (2) the sensed part (an elastic cylindrical beam of methacrylate), where some strain gauges are strategically attached. The elastic beam is clamped on both ends to the robotic fingertip. Figure 1e shows the sensor mounted on the fingertip of a BarrettHand, and Figure 1a shows a cross-section of the sensor and their dimensions.

In order to model the sensor, we assumed that the force, *F*, is applied in the plane, *Q* (see Figure 1b). The cover is clamped to the elastic beam at its midpoint, as shown in Figure 1c, and therefore, the applied force is transmitted to the elastic beam through this point. The applied force, *F*, can be decomposed into normal and tangential forces (*F_n_* and *F_t_*) relative to the surface of the metallic cover, as shown in Figure 1e. In addition, the normal force can be decomposed into Cartesian coordinates relatives to the axis (*X*, *Y*) associated with the elastic beam, as shown in Figure 1e. The magnitude and the direction of the applied normal force, *F_n_*, can be calculated with the following equations.

(1) $$\left|F_{n}\right|=\sqrt{F_{x}^{2}+F_{y}^{2}}$$

**Figure 2.**
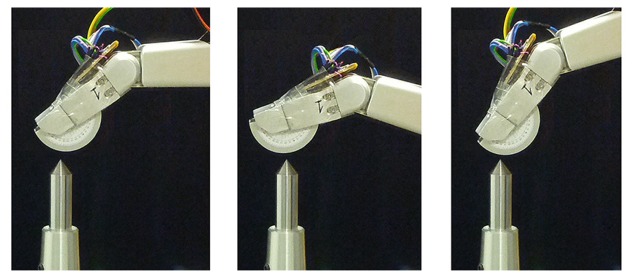
Contact point verification with different angles.

Knowing *F_x_* and *F_y_*, it is easy to obtain the region of the external part where the contact can be. This region is determined by angle *θ*; being *θ* = *arctg*(*F_x_*/*F_y_*). Therefore, the contact region on the surface of the cover (see Figure 1c) can be determined in polar coordinates, knowing *θ* and the metallic cover radius. In practice, the contact region will be the tangential segment to *F* belonging to the surface of the external part. As can be seen, the thinner the external part is, the smaller the possible contact region is. The sensor was verified by means of contact with a pointer, as shown in Figure 2, with different finger orientations and calculating *θ* from gauge readings.

On the other hand, the tangential force generates a moment on the *Z*-axis of the elastic beam. The magnitude of this force can be calculated as *F_t_* = *M_z_*/*r*, where *r* is the radius of the semi-cylindrical metallic cover.

**Figure 3.**
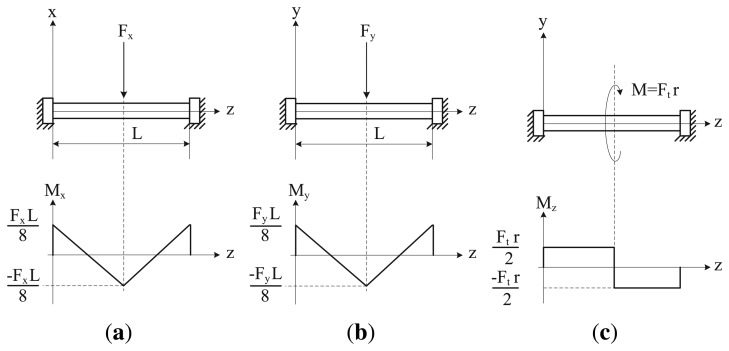
(**a**) Bending moment *M_x_*; (**b**) bending moment *M_y_*; (**c**) torsional moment *M_z_*.

**Figure 4.**
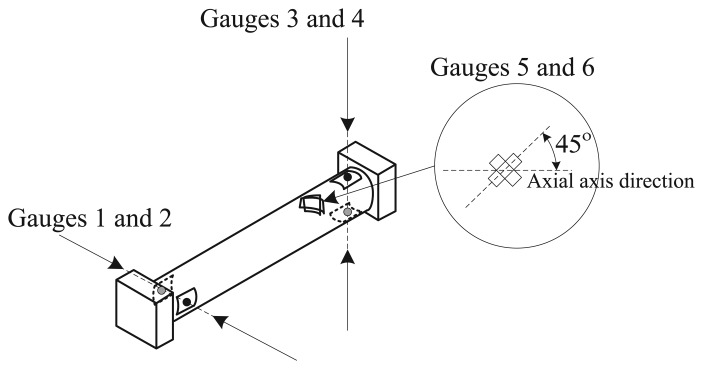
Location of the strain gauges. Gauges 1 and 2 are used to measure *M_x_*. Gauges 3 and 4 are used to measure *M_y_*, and gauges 5 and 6 are used to measure *M_z_*.

Figure 3 shows the bending moments (*M_x_*, *M_y_*) and the torsional moment, *M_z_*, transmitted to the elastic beam, caused respectively by *F_x_*, *F_y_* and *F_t_*. These transmitted moments can be measured by using strain gauges placed strategically on the elastic beam. Figure 4 shows the location of the strain gauges used to measure each transmitted moment. Bending moments *M_x_* and *M_y_* are measured by two pairs of gauges (1–2 and 3–4, respectively) placed in opposition at the ends of the beam, and the torsional moment, *M_z_*, is measured by two gauges (5–6) placed 45° with respect to the axial axis of the beam. With these configurations and an appropriate electrical connection to a Wheatstone bridge (two-gauge system), the superimposed effects can be canceled, measuring only the desired variable [33]. Therefore, the linear relation between the gauge voltage signals and the applied forces can be finally expressed by the following three equations.

(2) $$\begin{array}{ccc} V_{g12}=k_{x}\;F_{x}; & V_{g34}=k_{y}\;F_{y}; & V_{g56}=k_{t}\;F_{t} \end{array}$$

where *k_x_*, *k_y_* and *k_t_* are constants that must be calibrated and that determine the force sensor sensitivity (see Section 4.2).

## 3. Slip Detection Algorithm

When the surfaces of two objects come into slipping contact, some structural vibrations of high frequencies arise in the objects. This has been tested in previous works (e.g., [26]), and it will also be demonstrated in the present study.

In order to detect a slipping contact in real time, we have used an algorithm based on the discrete Fourier transform (DFT) of the strain gauge signals of the tactile sensor. This method allows us to detect structural micro-vibrations of high frequencies in real time and, therefore, slipping contacts.

It is well known that the DFT of a signal, *φ*, is defined by Equation (3).

(3) $$\begin{array}{cc} \Phi (k)=\overset{N-1}{\underset{n=0}{\text{∑}}}\varphi (n)e^{-i2\pi k\frac{n}{N}}; & k=0,\ldots ,N-1 \end{array}$$

where *N* is the number of samples used. Instead of evaluating Equation (3) directly, which requires *O*(*N*^2^) operations (*O* denotes an upper bound), we have used the Cooley-Tukey algorithm [34]. This algorithm is a fast Fourier transform (FFT) that computes the same results in *O*(*N* log*N*) operations (lower computational cost).

The power spectrum of Φ can be calculated as:

(4) $$Pw(k)=\frac{\Phi (k)\;\Phi^{*}(k)}{N}$$

where * denotes the conjugate.

The detection method is summarized as follows: (1) the FFT of the signal, Φ, is computed each *N* samples eliminating the DC component; (2) the power spectrum of Φ is calculated in every iteration, obtaining the peak value and its frequency; (3) the peak value of the power spectrum is multiplied by its frequency; and (4) finally, a slipping contact is detected when this later value exceeds a threshold defined empirically.

In order to detect slipping contact (associated with the microvibration of high frequencies) in real time, the sampling frequency was chosen to be *f_s_* = 1 kHz, and the FFT was computed with the last *N* = 64 samples of the strain gauges signals. This means that the time needed to detect slipping is *N*/*f_s_* = 64 ms, so we can consider that the slip detection is done in real time.

## 4. Experimental Results

The objectives of this section are to calibrate the sensor and to analyze its behavior in order to measure forces, locate the contact points and detect slipping. First of all, a brief description of the setup used for performing all experiments is introduced.

### 4.1. Experimental Setup

The force sensor was mounted on the fingertips of a robotic hand (BarrettHand). The hand was attached to a 6-Degrees of freedom(DOF) manipulator (model Stäubli RX-90). This manipulator was used to move the sensor toward an object and to apply a certain force. The motion control of this manipulator is not explained here, because it is not the objective of this study. The strain gauges used (model Kyowa KFG-02-120-C1) have a resistance of 120 ± 0.2 Ω and a gauge factor of 2.24 ± 1, and their dimensions are approximately a 1-mm square grid. Commercial strain gauge amplifiers (model Vishay BA660) were used for conditioning the gauge signals. All gauge signals were read with a computer by means of a commercial data acquisition card (model National Instruments(NI) PCIe-6363) and were processed in real time with the commercial data logging application, LabView. The sampling frequency for real-time data acquisition tasks was chosen to be 1 kHz.

### 4.2. Calibration of the Sensor

**Figure 5.**
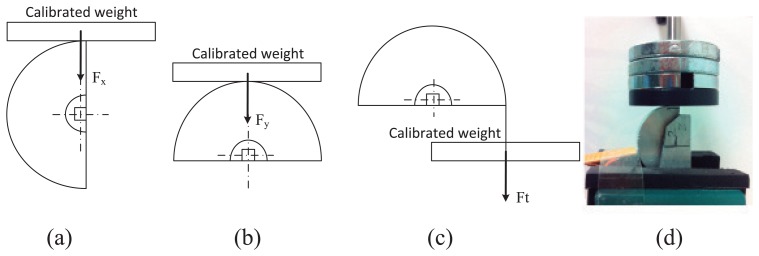
Scheme of the experiments carried out to calibrate the gauge signals: (**a**) setup configuration to calibrate the gauge signal that measures the force, *F_x_*; (**b**) setup configuration to calibrate the gauge signal that measures the force, *F_y_*; (**c**) setup configuration to calibrate the gauge signal that measures the force, *F_t_*; (**d**) real calibration example.

The three gauge signals of the sensor were calibrated by using the setup shown in Figure 5. Forces *F_x_*, *F_y_* and *F_t_* were applied on the sensor by using calibrated weights.

**Figure 6.**
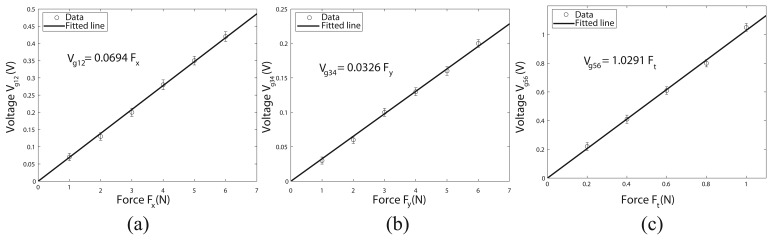
Calibration of the gauge signal used to measure the force, *F_t_*.

As previously said in Section 2, strain gauges 1 and 2 measure the applied force, *F_x_*. Figure 6a shows the voltage given by these gauges for different values of the applied force, *F_x_*. Several experiments were done for each value of the applied force (loading and unloading). The range of the voltage values obtained are represented with vertical lines. A straight line with the independent term equal to zero has been fitted to the data by means of the square minimum method, obtaining the calibration value: *V_g_*_12_ = 0.0694*F_x_*. The hysteresis, defined as the output variation for a same input value, depending on the direction (increasing or decreasing) of the input variable, can be calculated by the following expression [16]:

(5) $$\%\text{hysteresis}=100\frac{\text{MOD}}{\text{FSO}}$$

where MOD is the maximum output difference for the same input and FSO is the full-scale output. The hysteresis was 6% in this case.

The other two gauge signals, which measure the forces, *F_y_* and *F_t_*, were calibrated following the same previous procedure. Figure 6b, c shows, respectively, the voltage given by gauges 3–4 and gauges 5–6 for different values of the applied forces, *F_y_* and *F_t_*. Straight lines were fitted in each case, obtaining the calibration values: *V_g_*_34_ = 0.0326*F_y_*; *V_g_*_56_ = 1.0291*F_t_*. The hystereses in these cases were 4.8% and 5%, respectively.

From the results, the different sensitivity of the signals can be observed. While the sensitivity of the signals, *V_g_*_12_ and *V_g_*_34_, are of the same order, the sensitivity of the signal, *V_g_*_56_, is significantly higher. This is mainly caused by small misalignments of the strain gauges, but it is not a critical issue that prevents the operation of the sensor, as it is solved by the calibration.

The resolution is defined as the smallest change detected in the measured variable. For this force sensor, the resolution was 0.12 *N* for *F_x_* and *F_y_* and 0.01 *N* for *F_t_*. The sensor has been subjected to loads below 7 *N* for *F_x_* and *F_y_* and 1 *N* for *F_t_*. In order to avoid damaging the sensor, the authors do not recommend exceeding 10 *N* for *F_x_* and *F_y_* and 1.5 *N* for *F_t_*.

### 4.3. Slipping Test

**Figure 7.**
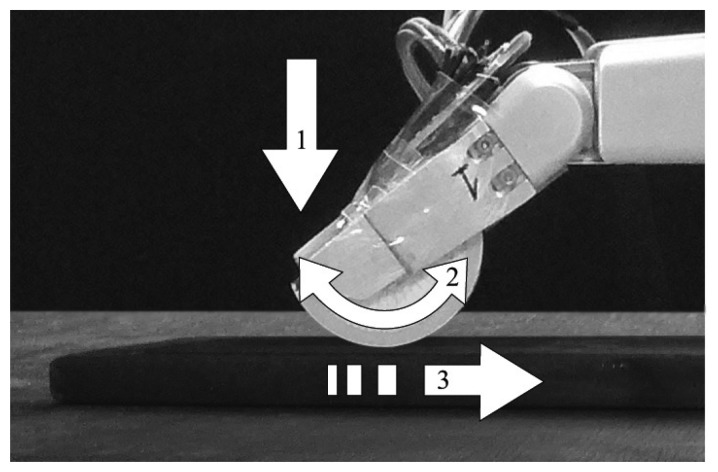
Experimental setup with our sensor.

The objective of the test was to demonstrate the effectiveness of the tactile force sensor in measuring the contact forces (*F_n_*, *F_t_*), locating the contact points and detecting slipping. A steel table (flat surface) was chosen to be the contact object. The test was performed according to the following sequence: (1) the fingertip was moved toward the table until the sensor came into contact with it; (2) the finger was rotated, allowing the semi-cylindrical surface of the sensor to roll on the table (rolling contact); (3) the finger was linearly moved, causing the tactile sensor to slip on the surface of the table (slipping contact); and (4) the finger was stopped. Figure 7 illustrates the aforementioned sequence.

**Figure 8.**
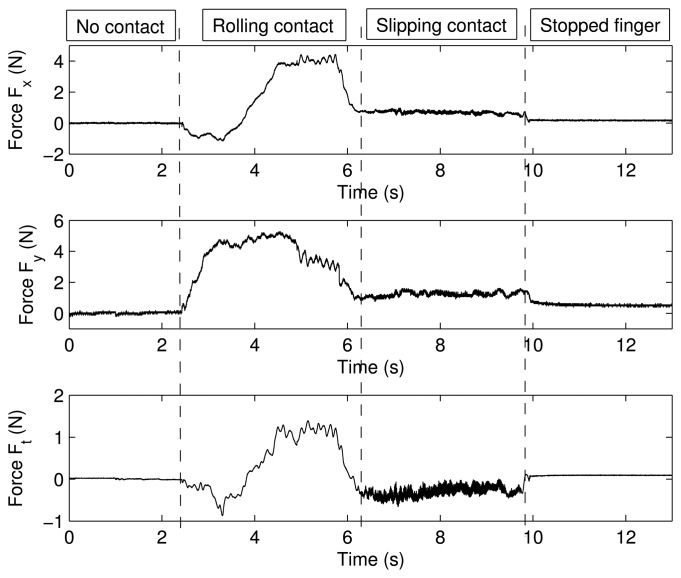
Forces *F_x_*, *F_y_* and *F_t_* as a function of time.

**Figure 9.**
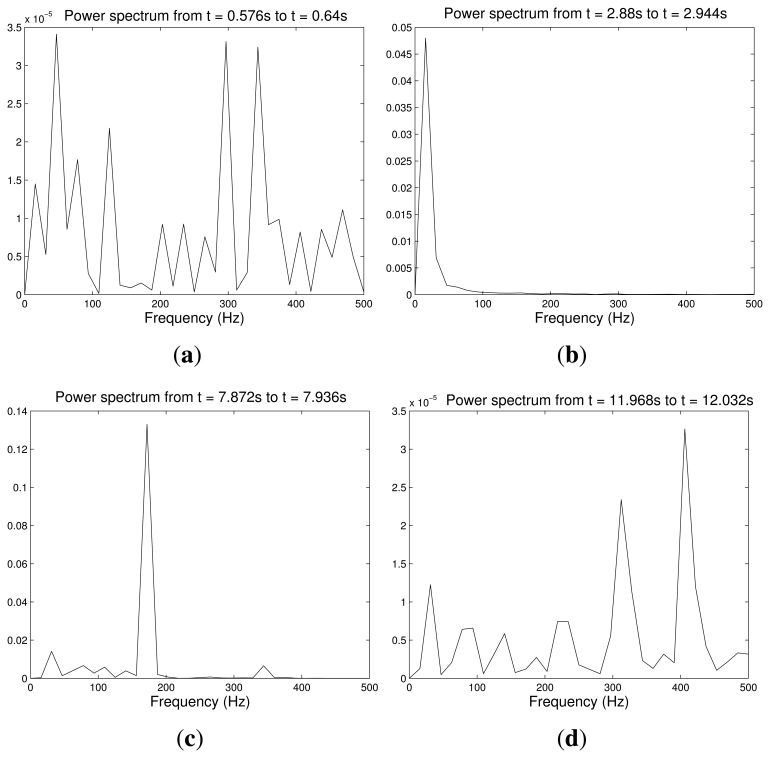
Power spectrum: (**a**) before contact; (**b**) rolling contact; (**c**) slipping contact; (**d**) stopped sensor.

**Figure 10.**
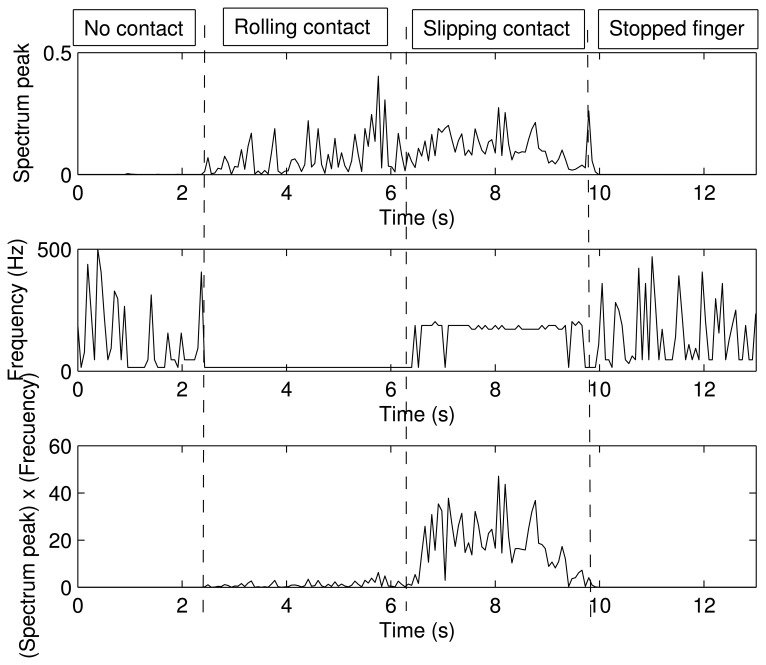
Spectrum peak and its frequency as a function of time.

Figure 8a shows the forces, *F_x_*, *F_y_* and *F_t_*, measured by the strain gauges during the experiment, and Figure 8b shows the magnitude and the direction of the contact force, *F_n_*, calculated according to Equation (1). It is seen that the sensor came into contact with the table at *t* = 2.43 s. From *t* = 2.43 s to *t* = 6.20 s, the cylindrical surface of the sensor rolled on the table. From *t* = 6.20 s to *t* = 9.8 s, a slipping contact occurred. Finally, the finger stopped its movement at *t* = 10 s. Structural micro-vibrations of high frequencies caused by the slipping contact appeared most clearly in the signal that measured the tangential force, Ft (this fact is obvious if we take into account that Ft is a friction force in this particular case). Figure 9 shows the power spectrum at four different instants of the signal that measured *F_t_*: (a) before contact; (b) rolling contact; (c) slipping contact; and (d) stopped finger. These spectrums represent the frequency content of this signal up to the Nyquist frequency. Note that the slip vibration frequency is about 180 Hz. Figure 10 (Top, Middle) shows, respectively, the peak value of the power spectrum and its frequency at each iteration (*N* = 64 samples, *i.e.*, each 64 ms), and Figure 10 (Bottom) shows the product of both signals. It is observed that the slipping contact can be detected when this product exceeds a certain threshold (after performing many experiments, this threshold was chosen as 10). Note that high frequencies caused by noise in the signal (from *t* = 0 s to *t* = 2 s and from *t* = 10 s to *t* = 13 s, as shown in Figure 10 (Middle)) and high spectrum peaks caused during the rolling contact, as shown in Figure 10 (Top), are ignored by this method, detecting high frequencies associated with high spectrum peaks, two conditions which only appear with slipping contacts.

**Figure 11.**
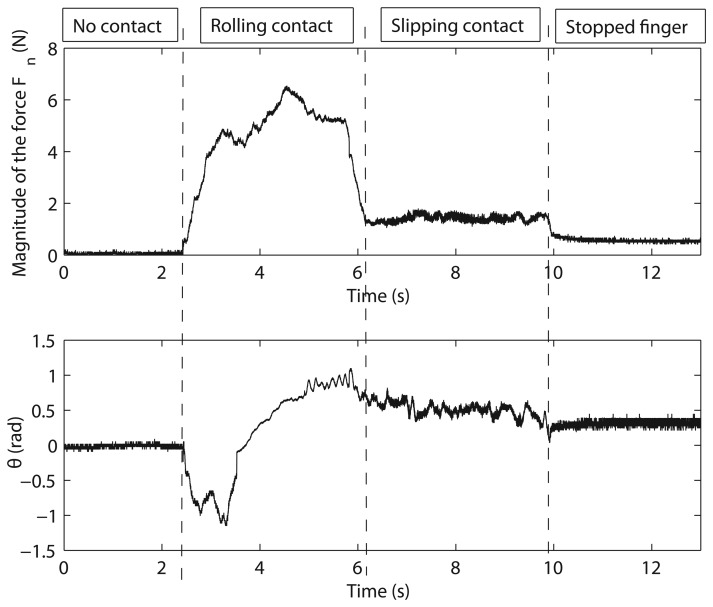
Magnitude and direction of the force, *F_n_*.

Finally, as our sensor allows us to locate the contact point in polar coordinates (see Section 2), it is possible to know where the slip occurs. This information could be useful in order to avoid the loss of contact during manipulation when object movement due to slippage is close to the border of the finger. Figure 11 shows the location of the contact by means of *θ*. It can be verified that, because of the movement of the finger, the contact location changes during the rolling motion.

## 5. Slip Detection with other Sensors

In addition to previous analysis, in this section, we analyze if the same slip detection procedure can be applied to other sensors. The results are compared in Section 6 in order to evaluate the performance of our sensor with respect to other sensors. In particular, we have tested four different sensors systems based on structural deformation and one sensor system based on piezoresistive matrices.

### 5.1. Flexible Finger Gripper Acting as a Force Sensor

**Figure 12.**
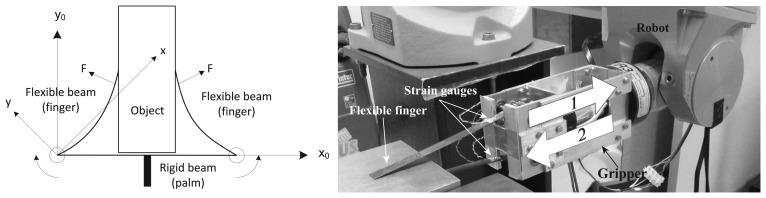
(**a**) Gripper scheme; (**b**) experimental setup with a flexible gripper.

As is demonstrated in [7], the flexible fingers of the gripper shown in Figure 12b can be used as force sensors. In particular, this gripper is an underactuated mechanism constituted by rigid parts (palm) and flexible parts (flexible fingers), as shown in Figure 12a. A pair of strain gauges is placed in opposition on the base of each elastic finger, as shown in Figure 12b; each pair is connected to a Wheatstone bridge that is wired to a Vishay BA660 strain gauge amplifier to measure the deflection and, therefore, the applied force.

#### Experimentation and Discussion

**Figure 13.**
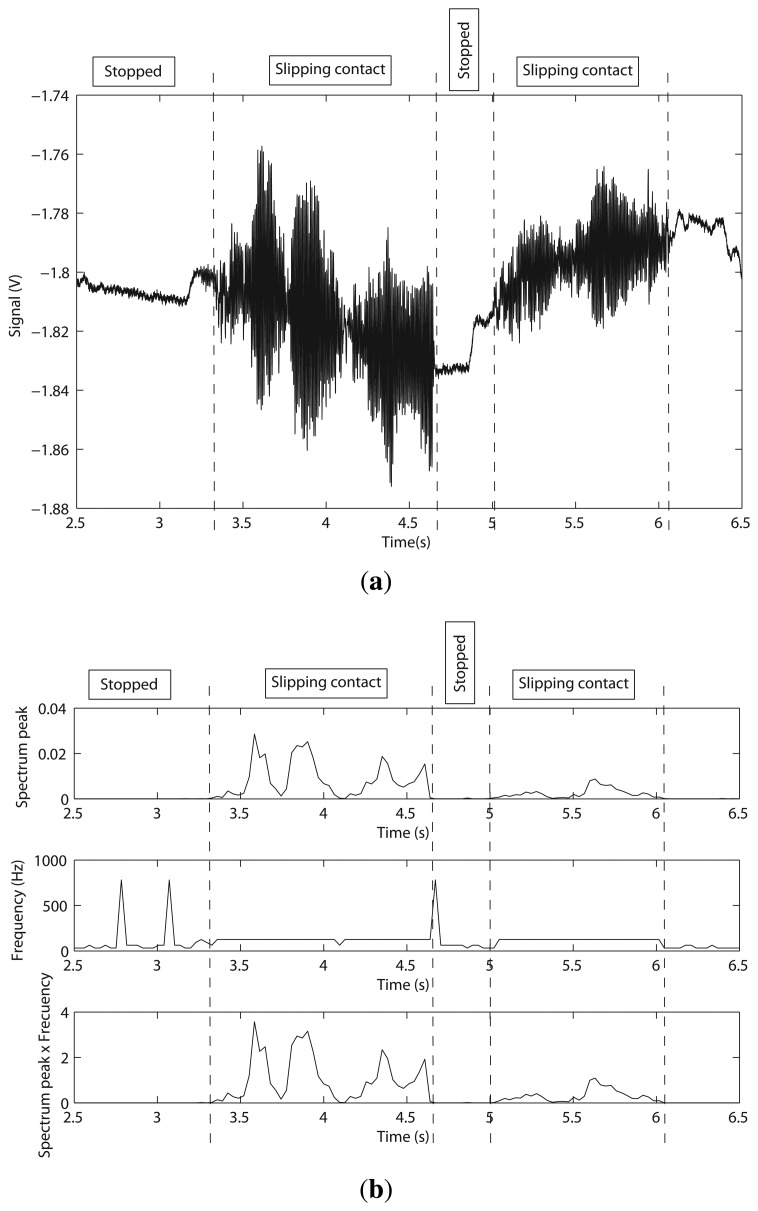
(**a**) Gauge voltage for finger 1 as a function of time; (**b**) spectrum peak and its frequency as a function of time (gripper).

The gripper was used as an end effector of a Stäubli RX90 robot, as shown Figure 12b. The contacted object was chosen to be a steel plate (flat surface), as shown in Figure 12b. The test was performed according to the following sequence: (1) the fingers were slipped on the surface of the plate; (2) the fingers were slipped in the opposite direction. Figure 13 shows the gauge signal of one of the fingers, measured by the strain gauges during the experiment. It is seen that the fingers slip in the plate from *t* = 3.24 s to *t* = 4.65 s. From *t* = 4.65 s to *t* = 5.01 s, the gripper kept slipping still. From *t* = 5.01 s to *t* = 6.06 s, the fingers slip in opposite directions. Finally, the gripper was stopped at *t* = 6.06 s.

Based on the algorithm described in Section 3, we looked for high frequency vibrations. After many experiments, the threshold to detect slipping was chosen as 0.25 V. Structural microvibrations of high frequencies caused by the slipping contact were detected, as can be seen in Figure 13b. In this system, the frequency of slipping vibration was detected at 125 Hz. We can conclude that the method proposed in Section 3 works for this sensor properly.

### 5.2. BarrettHand Torque Finger Sensor

**Figure 14.**
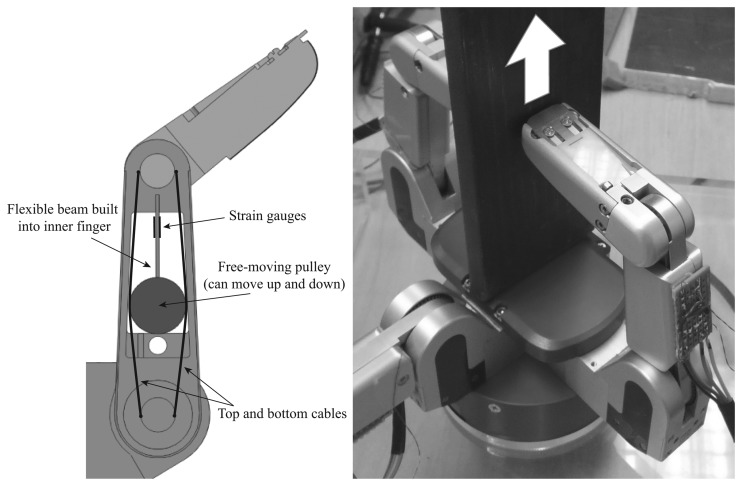
(**a**) A cutaway diagram of the finger reveals the internal strain gauges on the BarrettHand; (**b**) The experimental setup with the BarrettHand.

Each finger of a BarrettHand was mounted with a force sensing mechanism consisting of a flexible beam, a free-moving pulley, a pair of cables and two strain gauges, as is shown Figure 14a [5]. Basically, when a force is applied to the last phalange of the finger, the cables become tight, which moves the pulley, bending the flexible beam built into the inner finger. This deformation is measured by the strain gauges.

#### Experimentation and Discussion

**Figure 15.**
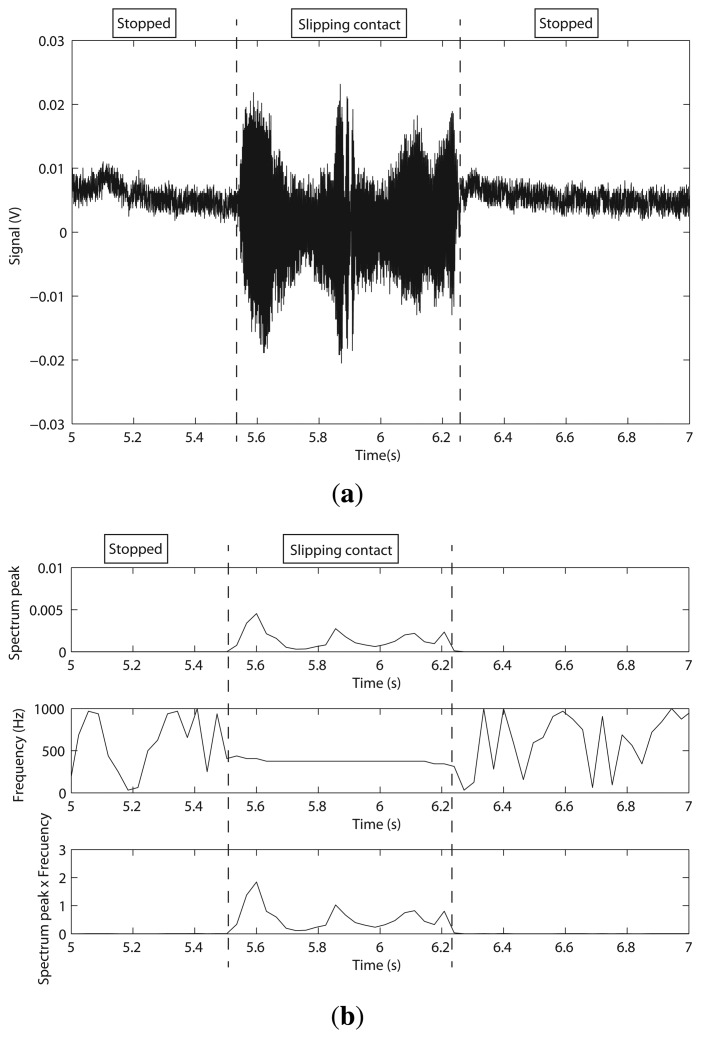
(**a**) Gauge signal finger 1 as a function of time; (**b**) spectrum peak and its frequency as a function of time (BarrettHand).

The BarrettHand was used again as an end effector of a Stäubli RX90 robot for these experiments. In the same way as in the previous experiment, the contacted object was chosen to be a steel plate, as shown in Figure 14b. The test was performed according to the following sequence: (1) the BarrettHand grasped the steel plate with two fingers; and (2) the fingers were slipped on the surface of the plate until they lost contact with the object. Figure 15a shows the gauge signal of one finger during the experiment. It is seen that the fingers slipped on the plate from *t* = 5.50 s to *t* = 6.25 s.

Structural microvibrations of high frequencies caused by the slipping contact were detected with this sensor, as we can see in Figure 15b. In the Barrett system, the frequency of slipping vibration was detected at 375 Hz. The frequency graph of Figure 15b shows that this system has a lot of high frequency noise; however, the detection method still works properly.

### 5.3. JR3 Six-Axis Force/Torque Sensor

**Figure 16.**
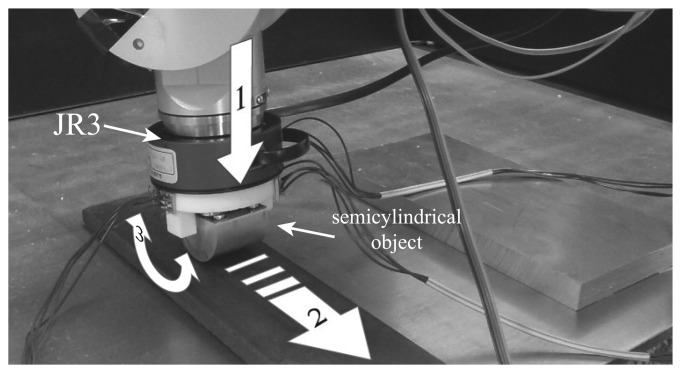
Experimental setup with JR3.

Manipulator robots usually have multi-axis force/torque sensors mounted on the wrist that are very useful in manipulation tasks. Usually, these sensors have strain gauges, like in the previously described sensors, to measure deformations in order to calculate the applied loads. We have used in this work the model 67M25A3 of JR3(Figure 16), which is able to measure *Fx* and *Fy*, with a standard measurement range of 100 *N* and a digital resolution of 0.025 *N*, and *Fz*, with a standard measurement range of 200 *N* and a digital resolution of 0.05 *N*.

#### Experimentation and Discussion

**Figure 17.**
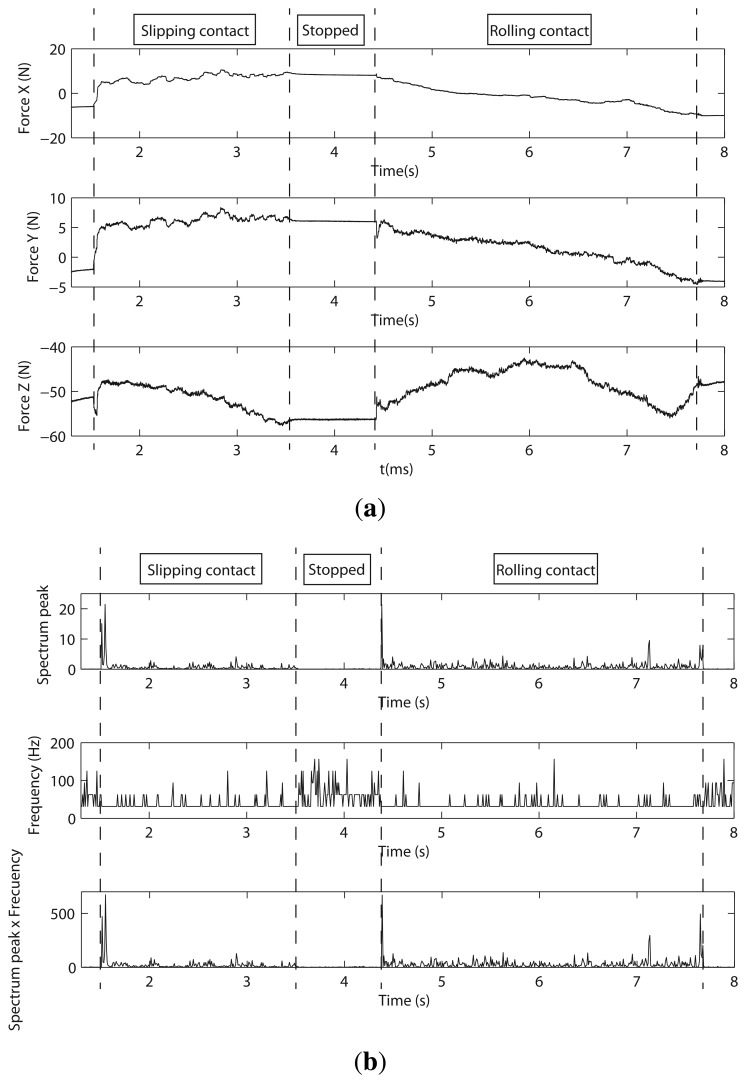
(**a**) The forces in sensor JR3 as a function of time; (**b**) spectrum peak and its frequency on *F_z_* as a function of time.

This sensor was mounted on the wrist of a Stäubli RX90 robot. As shown in Figure 16, it was necessary to add a semicylindrical object as a end-effector to perform the following experiment: (1) the semicylindrical object was moved toward the table until it came into contact with it; (2) the object was slipped on the surface of the table (slipping contact); and (3) the robot rolled the semi-cylindrical surface of the sensor on the table (rolling contact). Figure 17a. shows the forces, *F_x_*, *F_y_* and *F_z_*, measured during the experiment. It is seen that the object came into contact with the table at *t* = 0.82 s. From *t* = 1.53 s to *t* = 3.55 s, a slipping contact occurred. From *t* = 4.43 s to *t* = 7.75 s, the cylindrical surface of the object rolled on the table. Finally, the robot stopped its movement at *t* = 7.75 s.

Figure 17b shows the spectrum analysis of signal *F_z_*, which, based on its magnitude, was considered as the best one for detecting slipping. However, structural microvibrations of high frequencies were not detected. Basically, this is because of the relative amplitude and frequencies of the vibration modes when a slip occurs, which is not different in the rolling contact case. We have concluded that this is due to the high rigidity of this type of sensor.

### 5.4. Piezoresistive Matrix Sensor

Piezoresistive touch sensors are made of materials whose resistance changes with force/pressure. The tactile sensor, DSA 9210 (Weiss-Robotics), is especially designed to be used in the fingertips of robot hands and distinguishes itself through its specially curved surface. DSA 9210 is equipped with a sensing matrix with 70 sensing cells (taxels) and a spatial resolution of 3.4 mm. This matrix is covered by rubber, which, similar as is shown in [26], vibrates during the slipping of the object. The tactile data during the experiments were acquired with the DSACOM-32-M Controllerwith a 10-ms sampling time.

#### Experimentation and Discussion

**Figure 18.**
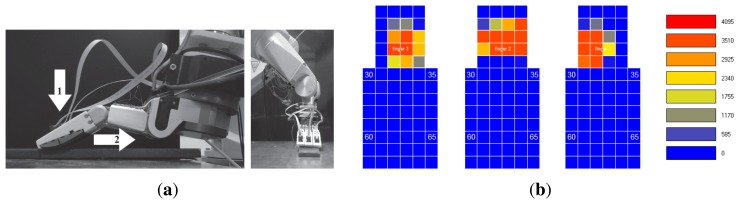
(**a**) Experimental setup with a matrix sensor; (**b**) graphical data of the three matrix sensor.

As can be seen in Figure 18a, the BarrettHand with three DSA 9210 sensors, one per fingertip, was used again as an end effector of a Stäubli RX90 robot for these experiments. The same steel plate as previous experiments was contacted, as shown in Figure 18. The test was performed according to the next sequence: (1) the three fingers of the BarrettHand were moved independently towards the plate until the contact force for each sensor reached a threshold of 250 kPa (Figure 18b represents graphically the tactile data from each sensor after this step); and (2) the whole hand was moved, allowing for the slipping of the fingertips on the surface of the plate until they lost contact with the object. The FFT algorithm, with *N* = 16, was run on each taxel of the three sensors. The taxel with the highest frequency was used to detect slipping.

**Figure 19.**
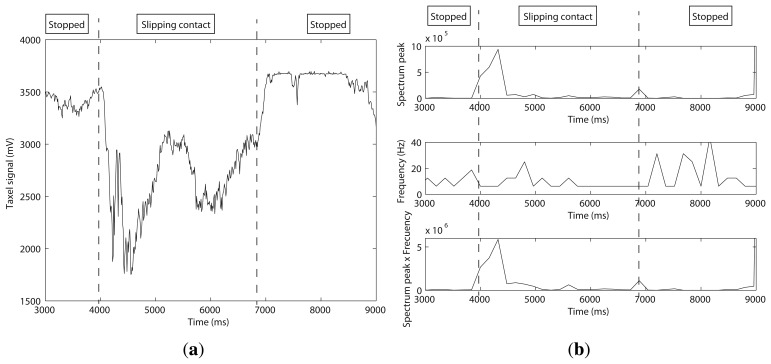
(**a**) The forces in the matrix sensor as a function of time; (**b**) spectrum peak and its frequency on a taxel as a function of time.

Figure 19a shows the contact data for this particular taxel. It is shown that the fingers slipped on the plate from *t* = 4, 000 ms to *t* = 7, 000 ms. Figure 19b shows the resulting DST analysis for this particular taxel. It can be observed that there is a high value of the spectrum peak at the beginning of the slipping, but the frequency analysis does not reveal a big difference between slipping and not slipping. In this case, the sampling rate of 10 ms does not allow us to determine if high frequencies exist, it being necessary to reduce this. A possible solution, planned as a future work, would be acquiring less taxels and not the whole matrix, which could improve the sampling rate.

## 6. Conclusions

We have presented a new force tactile sensor based on resistive strain gauges. The sensor can be mounted in the fingertips of a robotic hand easily, and is simple, durable and low cost. Its usefulness for measuring and locating forces has been confirmed with experimental tests in Section 4.2 We have also presented a simple algorithm, based on DFT, that allow us to detect slipping with our sensor in 64ms as presented in Section 4.3.

In addition, we have analyzed the usefulness of our method in other sensors based on strain gauges and piezoresistive matrices. Table 1 shows a comparison based on the results obtained via the experimentation depicted in previous Sections. As shown in the experiments the slipping detection algorithm perfectly works in most of the sensors tested, with better performance in those constituted by flexible parts, which demonstrate the feasibility of using these types of sensors for grasping and manipulation tasks. We can conclude the following statements:

- Slip detection based on DFT analysis has been successfully demonstrated with sensors based on flexible parts.
- Experimentation showed that sensors with high rigidity are not suitable for slipping detection because the amplitude and frequencies of their vibration modes cannot be distinguishable in slipping or rolling contacts. This is the case of the BarrettHand finger torque sensor and, above all, the force/torque wrist sensor.
- To detect microvibrations high sampling rate is required. Complex sensors, like the matrix-based sensors, adjust the sampling rate according to the number of active cells. One possible solution to use these sensors for slipping detection via high frequencies detection is to reduce the number of active cells.
- Our sensor gathers the necessary specifications in order to detect slippage with a simple but effective design.

## Figures and Tables

**Table 1.** Sensor comparison.

**System**	**Technology**	**S. Rate**	**Noise**	**Installation Zone**	**Slip Detection**
Our Sensor	Strain gauges	1 ms	Medium	Hand fingertips	Good
Flex. Finger	Strain gauges	1 ms	Medium	Robot end effector	Good
BH Sensor	Strain gauges	1 ms	High	Hand fingers	Regular
JR3 sensor	Strain gauges	1 ms	High	Robot wrist	Bad
Matrix Sensor	Piezoresistive	10 ms	Low	Hand fingertips	Bad
